# Indium droplet formation in InGaN thin films with single and double heterojunctions prepared by MOCVD

**DOI:** 10.1186/1556-276X-9-334

**Published:** 2014-07-04

**Authors:** Yung-Sheng Chen, Che-Hao Liao, Chie-Tong Kuo, Raymond Chien-Chao Tsiang, Hsiang-Chen Wang

**Affiliations:** 1 Graduate Institute of Opto-Mechatronics, National Chung Cheng University, 168 University Rd., Min-Hsiung, Chia-Yi 62102, Taiwan; 2 Institute of Photonics and Optoelectronics, National Taiwan University, Taipei 10617, Taiwan; 3 Department of Physics, National Sun Yat-sen University, 70 Lienhai Rd., Kaohsiung 80424, Taiwan; 4 Department of Chemical Engineering, National Chung Cheng University, Chia-Yi 62102, Taiwan; 5 Advanced Institute of Manufacturing with High-tech Innovations (AIM-HI), National Chung Cheng University, 168 University Rd., Min-Hsiung, Chia-Yi 62102, Taiwan

**Keywords:** Indium droplet, Heterojunction structure, InGaN, Metal-organic chemical vapor deposition

## Abstract

Indium gallium nitride (InGaN) samples with single heterojunction (SH) and double heterojunction (DH) were prepared using metal-organic chemical vapor deposition. SH has a layer of InGaN thin film (thicknesses, 25, 50, 100, and 200 nm) grown on an uGaN film (thickness, 2 μm). The DH samples are distinguished by DH uGaN film (thickness, 120 nm) grown on the InGaN layer. Reciprocal space mapping measurements reveal that the DH samples are fully strained with different thicknesses, whereas the strain in the SH samples are significantly relaxed with the increasing thickness of the InGaN film. Scanning electron microscopy results show that the surface roughness of the sample increases when the sample is relaxed. High-resolution transmission electron microscopy images of the structure of indium droplets in the DH sample indicate that the thickness of the InGaN layer decreases with the density of indium droplets. The formation of these droplets is attributed to the insufficient kinetic energy of indium atom to react with the elements of group V, resulting to aggregation. The gallium atoms in the GaN thin film will not be uniformly replaced by indium atoms; the InGaN thin film has an uneven distribution of indium atoms and the quality of the epitaxial layer is degraded.

## Background

(Al, Ga, In) N material systems have been extensively investigated because of their potential applications in light-emitting diodes (LEDs), laser diodes, and photodetectors [1-6]. Indium gallium nitride (InGaN) has high absorption, broad spectral coverage, and radiation hardness, and its alloys have emerged as new solar cell materials. InGaN is used for the conversion of sunlight in the visible range into electrical power. Thus, the growth of high-quality InGaN thin films with indium content higher than 20% has become important [7-11]. Given the large lattice mismatch (11%) between GaN and InN, phase separation occurs when the thickness of the InGaN film is larger than the critical value, which is <60 nm for indium content higher than 20% [12-16]. InGaN thin films are applied to solar energy materials with an optimum thickness of about 100 nm [17]. Although thick InGaN film is beneficial for absorption of more sunlight, this absorption and conversion efficiency will substantially be reduced. The photoelectric conversion efficiency will be reduced, and the recombination rate of the photoexcited carrier will improve because of the film thickness [18]. The thickness of the InGaN film (100 nm) is greater than its critical value in terms of film growth with high indium content because of the mismatch of GaN and InGaN in the crystal lattice; the strain is accumulated until the film is fully strained. Subsequently, the film is fully relaxed and has many defects [19]. Studies on the latest applications of InGaN thin films in solar energy have revealed that InGaN is applied to solar energy materials to increase the indium content. However, high indium content generates several indium-rich clusters, which, in turn, generate several defects and degrade the film quality [20]. The strain induced by the heterostructure within the critical thickness causes the indium-doped InGaN thin film to increase the indium content by reducing the generated indium droplets. Exceeding the thickness of the InGaN thin film to its critical value increases the average indium content by relaxing the indium droplet structure. Although InGaN thin films have been extensively grown and widely studied, the composition and distribution of InGaN film, as well as the energy gap distribution, are yet to be elucidated. The structures of indium droplets applied in the overlying p-type GaN film grown at high temperature in p-i-n heterostructures also remain unclear [21-23]. The reduction in uniformity and smoothness of the entire indium-doped InGaN thin film and the presence of irregular luminescent bands are caused by the aggregation of indium atoms, which reduces the overall indium content of the thin film, and consequently the luminescent quality [24]. The other cause is the existence of defects around the indium droplets, which increases the density of the defects [25]. Thus, the growth of high-quality InGaN thin film remains a challenge. In this study, we demonstrated the growth of SH and DH samples on *c*-plane sapphire substrates by a two-step temperature-dependent growth of InGaN layers. X-ray diffractometry (XRD) and energy dispersive spectrometry (EDS) were used to calculate the indium contents. The crystallization quality and strain distribution of the samples were analyzed through XRD measurements. Temperature-dependent photoluminescence results showed the dependence of the exciting confinement on the indium contents and the width of the barrier layer. The growth of SH and DH samples was observed based on the optimal barrier layer conditions. The SH and DH structures of the samples were verified by high-resolution transmission electron microscopy (HRTEM).

## Methods

**Figure 1:**
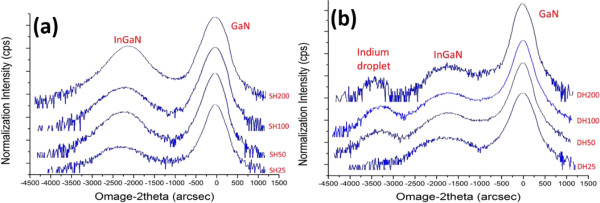
Structures of the SH and DH samples.

All samples were grown by metal-organic chemical vapor deposition (MOCVD). The InGaN/GaN SH samples were prepared as follows. First, a 2-μm undoped GaN layer was grown on (0001) *c*-plane sapphire substrate at 1,080°C. The InGaN layers were grown at a temperature of 700°C and thicknesses of 25, 50, 100, and 200 nm. DH samples were grown similar to the SH samples, but with an additional final GaN layer (thickness, 120 nm) grown at 900°C. Figure 1 shows the detailed structures of both samples. InGaN films with SH heterostructures (thickness, 25, 50, 100, and 200 nm) were named samples SH25, SH50, SH100, and SH200. The films with DH heterostructures (thickness, 25, 50, 100, and 200 nm) were named samples DH25, DH50, DH100, and DH200. The ω-2*θ* XRD scans determined the material composition of the samples. XRD was performed to obtain the reciprocal space mapping (RSM) and understand the strain condition in the samples. Indium content distribution can be calibrated based on the strain information. Scanning electron microscopy (SEM) measurements were conducted to compare the surface roughness of the SH and DH samples at different thicknesses. Energy dispersive X-ray spectrum (EDX) was obtained to yield the elemental distribution of the cross-section direction of the samples from transmission electron microscopy (TEM). Calibrated indium contents were confirmed by photoluminescence (PL) measurements through the excitation of the top and bottom portions of the substrate. The indium droplets within the InGaN films of the DH samples were observed by TEM.

## Results and discussion

**Figure 2:**
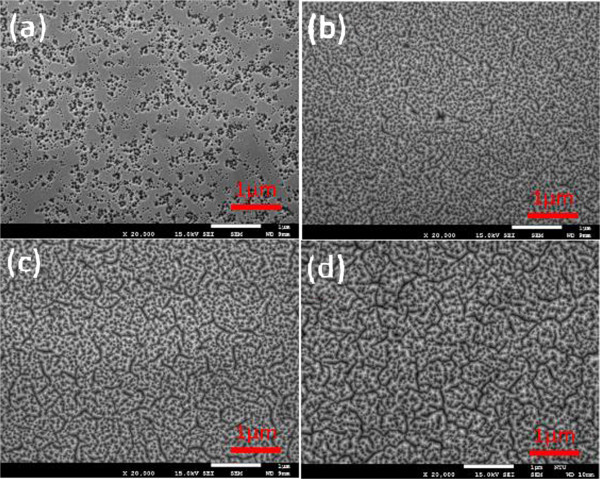
**XRD ω-2** ***θ *** **scanning results of the (a) SH and (b) DH samples.**

Figure 2a,b respectively shows the ω-2*θ* XRD measurements for the SH and DH samples. Two major peaks that correspond to GaN and InGaN films are observed (Figure 2a). Increasing the thickness of the InGaN film yields stronger XRD signal; the phase separation signal does not exist for this film. Figure 2b reveals a new peak, as well as those for the GaN and InGaN films. The new peak is induced by the indium droplets generated from the phase separation of the InGaN layer. Hartono et al. [26] attempted to grow InN directly on GaN, which resulted in the formation of indium droplets. They showed that the XRD measurements and the peak for indium droplets were consistent with our measurements. Formation of indium droplets in the InGaN layer of the DH sample was observed. The newly generated peak signal corresponds to the signal of these droplets (Figure 2b).

**Figure 3:**
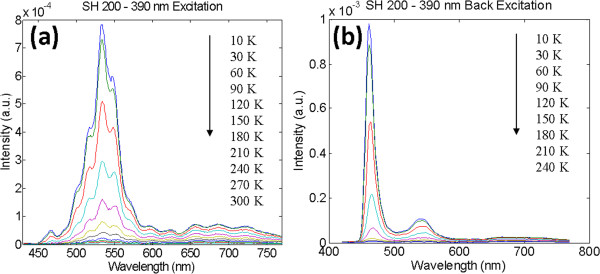
**RSM of (a) SH and (b) DH samples with 200-nm InGaN layer grown at 700°C.**

Figure 3 shows the RSM measurements of samples SH200 and DH200. The strain situation of these samples is discussed because of the marked RSM measurements for the sample with film thickness of 200 nm. Figure 3a indicates the existence of two different regions. The upper region corresponds to the space interfered by GaN on face (105), which is turned upside down; this region also presents the measurements of the fully strained GaN. The lower region represents the space interfered by InGaN; the distribution and the change in the strain inside the materials are simpler than that of DH200 because of the SH200 with SH heterostructure. In Figure 3a, in the part marked with InGaN, point S is fully strained, whereas point R is fully relaxed. Thus, the range from points S to R represents the growth of InGaN from fully strained to fully relaxed. Sample SH200 has exceeding critical thickness, with calculated concentration of 19% at point S and 27% at point R [27-29]. The growth changes from fully strained to fully relaxed because of the absence of GaN layer grown on the upper SH200 layer. Strain relaxation is caused by lattice mismatch between GaN and InGaN [30]. Figure 3b reveals that the positions of the upper portion of the GAN layer and the lower portion of the InGaN layer are similar and fully strained without any fully relaxed peak *Q*(*x*) as that of SH200. This result attributed to DH200 having a DH heterostructure; one more layer of GaN is grown over InGaN, which inhibits the InGaN layer to be in the fully relaxed state. Both layers are fully strained, and the InGaN layer is not relaxed based on the RSM diagram. These results yield better material characteristics for DH200. Most of the studies have pointed out the contribution of the strain relaxation from critical thickness of InGaN growth [28,31,32]. One of possible ways about the strain relaxation may come from higher temperature (900°C) GaN capping layer growth. Reed et al. demonstrated that the high-temperature (880°C) GaN cap results strain relaxation in the InGaN [33]. Choi et al. also reported that more indium diffusion in the InGaN interlayer after GaN capping layer is grown at high temperatures [34]. But we lack the confidence in this point since the current contents of this article do not have sufficient evidence to proof the contribution of higher temperature GaN capping layer growth.

**Figure 4:**
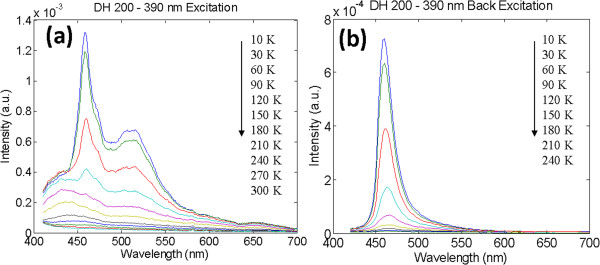
**SEM images (top view) of the SH samples. (a)** SH25. **(b)** SH50. **(c)** SH100. **(d)** SH200.

**Figure 5:**
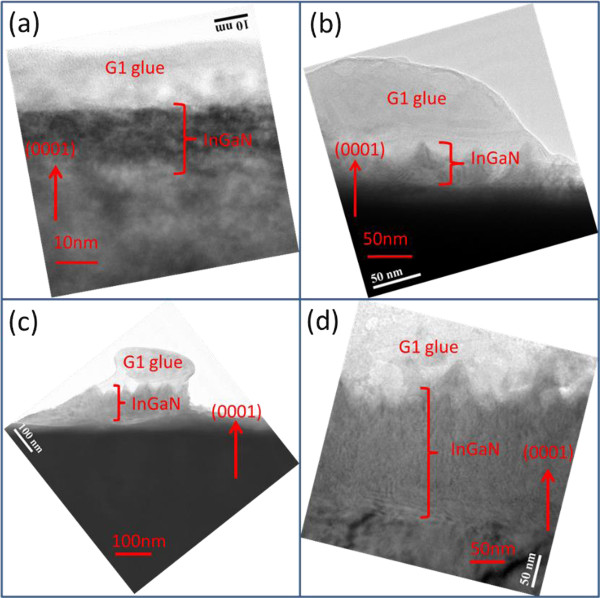
**SEM images (top view) of the DH samples. (a)** DH25. **(b)** DH50. **(c)** DH100. **(d)** DH200.

Figure 4a,b,c,d illustrates the SEM measurements for samples SH25, SH50, SH100, and SH200, respectively. The surface roughness of the SH heterostructure increases with film thickness because the crystal lattice of the GaN layer does not match with that of the InGaN thin film. The number of defects increases with the thickness of the InGaN thin film, thereby forming cracks. The existence of too many defects for restraint produces cracks on the surface and causes rose-like surface roughness [35]. The results show that the surface roughness of SH200 is the largest because of the most fully relaxed surface of this sample. Full relaxation produces the rose-like surface structure of SH200 [36,37], consistent with the RSM measurements.Figure 5a,b,c,d illustrates the SEM measurements for samples DH25, DH50, DH100, and DH200, respectively. The number of surfaces of the DH heterostructure increases with film thickness, producing a more coarse surface, with DH200 having the largest surface roughness. The surface roughness of samples DH25 and DH50 with film thicknesses within the critical value after growth is significantly lower than that of sample DH200. This result is attributed to the thickness of InGaN within the critical value, as well as the material that remains fully strained. Hence, the surface flatness of this material is superior to the other samples.

**Figure 6:**
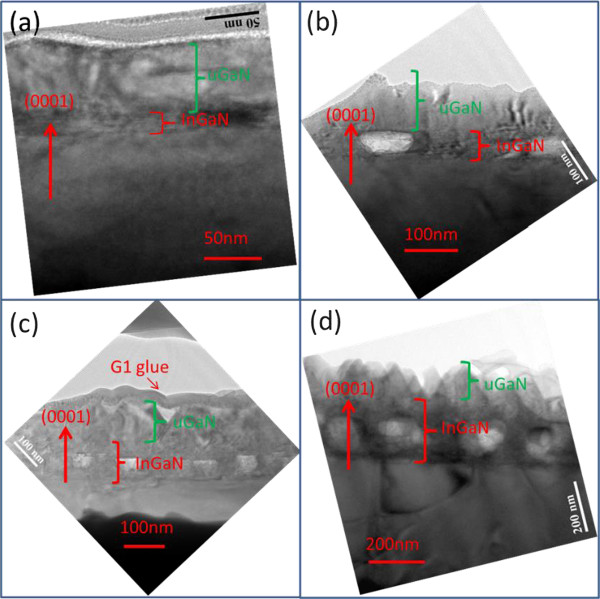
PL spectra of sample SH200 with excitation from the (a) top and (b) bottom portions.

Given the application of the growth of InGaN thin films to solar energy materials [38,39], this study focused on the optical properties of SH200 and DH200. Figure 6a,b illustrates the temperature-dependent PL measurements of SH200 given by the spectrograms of the front-side and back-side incidences, with the respective temperature ranges of 10 to 300 K and 10 to 240 K. The position of the measured front-side incidence is in front of the InGaN; the light-emitting band is about 530 nm (green light). By contrast, the position of the measure back-side incidence is at the back of InGaN; the light-emitting band is about 460 nm (blue light) with smaller signal at 530 nm. The positions are caused by the fully relaxed strain at the front of the InGaN thin film of SH200 and produces phase separation [40-42]. High indium content exists at the front of InGaN [34,43].

**Figure 7:**
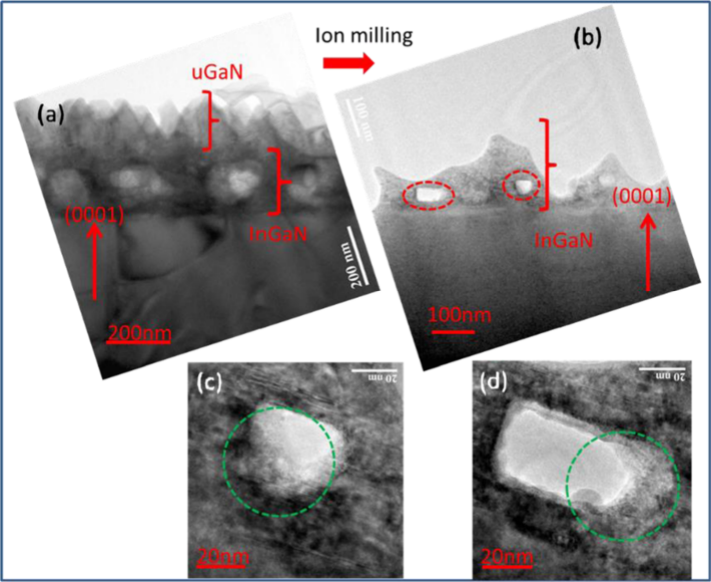
PL spectra of sample DH200 with excitation from the (a) top and (b) bottom portions.

Figure 7a,b shows the temperature-dependent PL measurements of DH200 given by the spectrograms of the front-side and back-side incidences; the respective temperature ranges are 10 to 300 K and 10 to 240 K. The spectrograms for the sample DH200 reveal that the light-emitting bands in the front-side and back-side incidences are concentrated at 460 nm. The results suggest that GaN layer exists at both sides of InGaN. The upper and lower parts of the InGaN layer are fully strained and have low indium contents. A light-emitting band at 510 nm exists at the front-side PL measurements [44-46] and emitted from the middle part of sample DH200. A fully relaxed intermediate layer is formed by the aggregation of indium droplets with relatively high indium contents, attributed to the changes in the DH200 strain. The fully strained condition was only included in the RSM measurements of sample DH200 (thickness, 200 nm). The RSM measurements of sample SH200 reveal two different indium concentrations. The PL measurements of the sample also show two different peaks at 460 and 530 nm, wherein the intensity at 460 nm is smaller. The aggregation of indium droplets occurs in DH200, which explains the visibility of light-emitting band at 510 nm in the PL measurements for the front-side incidence of DH200. The PL measurements of DH200 are consistent with the indium content based on the RSM measurements.

**Figure 8:**
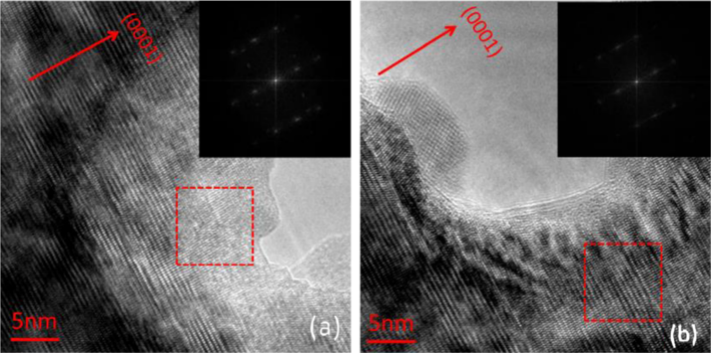
TEM images of samples (a) SH25, (b) SH50, (c) SH100, and (d) SH200.

**Figure 9:**
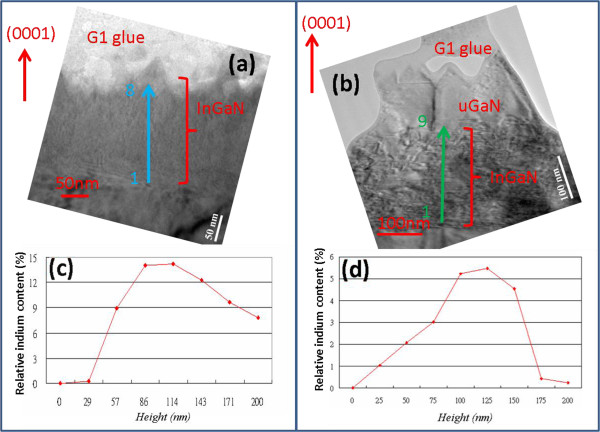
HRTEM images of samples (a) DH25, (b) DH50, (c) DH100, and (d) DH200.

Figure 8a,b,c,d illustrates the HRTEM images of samples SH25, SH50, SH100, and SH200, with cross-section directions. The red arrows in these images indicate the (0001) direction. The contrast in the HRTEM images is caused by the differences in atomic mass between indium-49 and gallium-31 [47]. The heavier atom of the material causes the darker part of one HRTEM image. The InGaN thin film surface is considerably flat and even (top panel, Figure 8a). Figure 8b,c,d implies that the difference between the peak-valley of the image contrast is larger than the other place below the G1 glue region. The range between the peak and the valley is the largest; hence, the roughness depth of SH200 and the thickness of the InGaN thin film are clearly observed (Figure 8d). This finding is consistent with the previous SEM measurements. Figure 9a,b,c,d shows the HRTEM images of samples DH25, DH50, DH100, and DH200, with cross-section directions. The green brackets represent the uGaN layer, whereas the red brackets represent the InGaN thin film. No holes were found in Figure 9a. The number of holes (Figure 9b,c,d) and the hole diameter increases with InGaN film thickness. Figure 9 reveals the existence of three more samples with holes aside from DH25 because of the indium droplets arising from the aggregation of indium [48-52].

**Figure 10:**
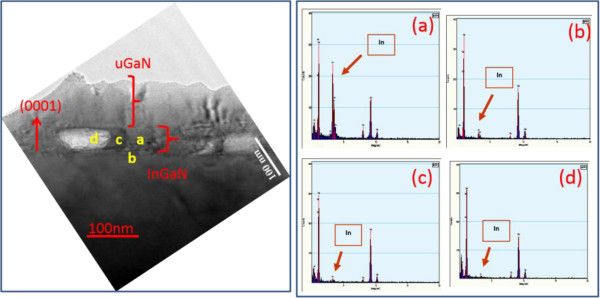
**Cross-section TEM images of DH200.** Before **(a)** and after ion milling (red rings as indium droplet) **(b)**, and the first **(c)** and second **(d)** holes (green rings) in (b).

**Figure 11:**
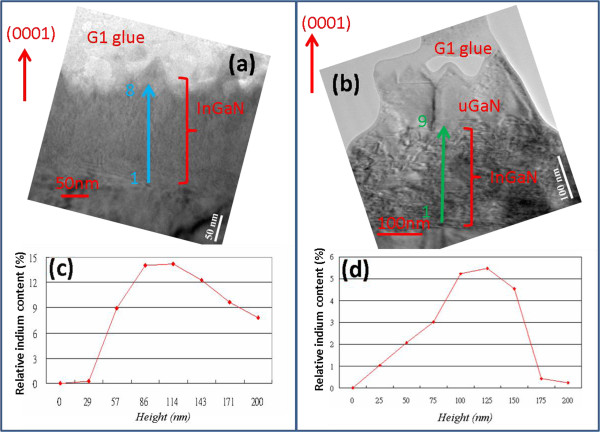
**Cross-section HRTEM images of DH200 from the (a) first and (b) second indium droplet holes.** The two dotted line squares are SAD areas that correspond to the FFT images.

We analyzed the formation mechanism of the indium droplets. Figure 10a,b,c,d shows the cross-section TEM images of DH200 and indium droplets (red and green rings), with (a) large scale images, (b) ion milling, (c) first indium droplet hole, and (d) second indium droplet hole, respectively. The holes are gathered in the middle of the samples. A few holes are located near the front and back parts of GaN; some thin films have holes. HRTEM was performed to determine the causes of the formation of indium droplets. Figure 11 shows the high-resolution images of the holes in sample DH200 through TEM with electron beam energy and voltage of 300 KeV. Figure 11a,b shows the cross-section HRTEM images of indium droplets (two green rings in Figure 10c,d) with first and second indium droplet holes. The insets are the diffraction patterns based on fast Fourier transform (FFT). Results reveal the existence of atomic lattice images of indium droplets around the hole [53-55]. The lattice pattern of the selected area of diffraction (SAD) shows that the direction of this pattern in the hole is different from that of the area without holes. From the formation of indium droplet in the hole, the crystal lattice in this area is the overlapping diffraction lattice pattern of InGaN and In atom aggregations (inset of Figure 11a). The diffraction lattice pattern around the outer circumference of the hole corresponds to InGaN/GaN (inset of Figure 11b), with significant differences [56]. Reducing the thickness of the InGaN film decreases the number of holes, consistent with the XRD measurements. No indium droplet peak is found in the ω-2*θ* curve of DH25.

**Figure 12:**
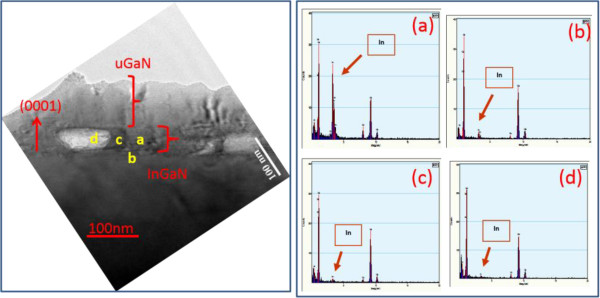
EDX measurements (a,b,c,d) of four points a to d (left image) in sample DH50.

The indium droplet images without holes were analyzed by EDX. Figure 12(a) illustrates the cross-section HRTEM images of DH50; Figure 12b,c,d,e shows the four-point EDX profiles. Table 1 tabulates the results from the EDX measurements. The measuring positions are the area not thinned out in the hole of the InGaN thin film (point a), in the InGaN thin film (point b), at the back and close to the InGaN thin film (point c), and in the hole of the indium droplet (point d). The respective measured indium contents are 28 at point a (93%), 4 at point b (42%), 1 at point c (41%), and 0 at point d (29%). Almost no indium is found at points c and d because the ion mill was used to thin the sample until a thickness of 100 nm is achieved while preparing the sample. The structure of the indium droplet is more fragile than that of the InGaN thin film; the sample quickly thins out with the ion mill and renders voids [57]. Hence, the presence of voids in the InGaN film corresponds to that of the indium droplet [58-60].

**Table 1:** EDX data of four points (labeled a to d) in sample DH50

**Element**	**Point a (%)**	**Point b (%)**	**Point c (%)**	**Point d (%)**
N (K)	44.64	44.46	59.80	56.41
Ga (K)	26.41	51.10	38.78	43.29
In (L)	28.93	4.42	1.41	0.29

**Figure 13:**
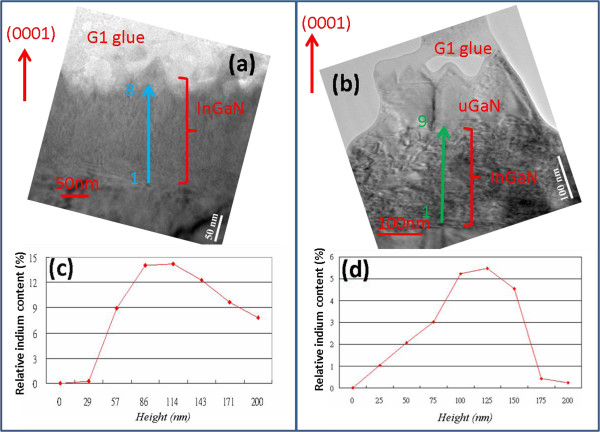
**TEM images.** TEM images of **(a)** SH200 and **(b)** DH200; EDX results of **(c)** SH200 from the blue line in (a) and **(d)** DH200 from the green line in (b).

Figure 13 shows the indium contents at different points (bottom-up) of the InGaN layer for samples SH200 and DH200; the contents were obtained from the EDX line scanning results. The red arrow in Figure 13 indicates the (0001) direction from the substrate. Figure 13a,c shows the EDX line scans and the indium content profile of SH200; Figure 13b,d represents sample DH200. Values along the direction of the blue arrow were measured at eight EDX points (Figure 13a). The values in the middle are high, whereas those on both sides are small (Figure 13c). EDX measurements reveal that the proximity to the upper part increases the indium content for SH200. Figure 13c, however, reveals that the proximity to the upper part of SH200 reduces the indium content. Terminating the growth of SH200 leaves the MOCVD machine in the hydrogen atmosphere at a high temperature for several minutes, which considerably affects the InGaN layer surface of SH200. A transient high-temperature environment causes thermal annealing that decreases the indium content close to the InGaN layer surface [61,62]. The indium content profile of the InGaN layer of sample DH200 is obtained from the EDX measurements (9-point, bottom-up, marked with green arrow; Figure 13d). This content is high at the middle and low on both sides, consistent with our XRD and PL measurements of DH200. The EDX line scanning results show that the indium contents in the upper and lower parts of the InGaN layer of DH200 are lower, whereas those in the intermediate layer is very high. The intermediate layer indicates the existence of indium droplets [63-65]. High-concentration indium exists at the intermediate layer as indium droplets, instead at the InGaN layer as a uniform thin film.

## Conclusions

We analyzed the nanostructures of the samples with SHs and DHs through HRTEM and SEM. HRTEM measurements reveal that the thickness of the InGaN film decreases with the density of the indium droplets. The surface roughness of the samples increases upon relaxation in these materials based on the SEM measurements. RSM and PL measurements indicate the variations of strain and indium composition of the InGaN layer in the samples. The distribution of indium content in the front and back sides of the InGaN layer was obtained from DH200. The change in the strain at both sides of the InGaN and the intermediate layers for the same sample was discussed. The SEM measurements indicated that increasing the thickness of the sample affects the strain relaxation of the sample. Differences exist between the strain relaxations of the SH and DH samples in the InGaN thin film. The indium droplets reduced the quality of the film by phase separation and poor electrical performance. EDX experimental results for samples SH200 and DH200 revealed that the thickness of the InGaN layer decreased with the density of the indium droplets. Thus, the thickness of the InGaN layer should be increased to avoid the formation of indium droplets. Unfortunately, a thin layer is insufficient for proper absorption in solar cells.

## Competing interests

The authors declare that they have no competing interests.

## Authors’ contributions

YSC and CHL performed the experiments and fabricated the samples. HCW coordinated the project. YSC performed the XRD, RSM, and TEM measurements. CHL performed the SEM, temperature-dependent PL, and EDX measurements. CTK, RCCT, and HCW drafted the paper. All the authors read and agree the final version of the paper.

## References

[B1] Nakamura S Fasol G The Blue Laser Diode GaN Based Light Emitters and Lasers 1997 New York: Springer SPIN 10543597

[B2] Chiou YZ Su YK Chang SJ Lin YC Chang CS Chen CH InGaN/GaN MQW p–n junction photodetectors Sol State Elect 2002 9 2227 2229 10.1016/S0038-1101(02)00230-7

[B3] Huh C Lee KS Kang EJ Park SJ Improved light-output and electrical performance of InGaN-based light-emitting diode by microroughening of the p-GaN surface J Appl Phys 2003 9 9383 9385 10.1063/1.1571962

[B4] Huang FW Sheu JK Lee ML Tu SJ Lai WC Tsai WC Chang WH Linear photon up-conversion of 450 meV in InGaN/GaN multiple quantum wells via Mn-doped GaN intermediate band photodetection Opt Exp 2011 9 1211 1218 10.1364/OE.19.0A1211 22109617

[B5] Rivera C Pereiro J Navarro Á Muñoz E Brandt O Grahn HT Advances in group-III-nitride photodetectors Ope Elect Elect Eng Jour 2010 9 1 9 10.2174/1874129001004010001

[B6] Ee YK Biser JM Cao W Chan HM Vinci RP Tansu N Metalorganic vapor phase epitaxy of III-nitride light-emitting diodes on nano-patterned AGOG sapphire substrate by abbreviated growth mode IEEE J Sel Top Quantum Electron 2009 9 1066 1072

[B7] Morkoç H Nitride Semiconductors and Devices 1999 New York: Springer

[B8] Mauder C Reuters B Wang KR Fahle D Trampert A Rzheutskii MV Lutsenko EV Yablonskii GP Woitok JF Chou MMC Heuken M Kalisch H Effect of indium incorporation on optical and structural properties of m-plane InGaN/GaN MQW on LiAlO_2_ substrates J Cryst Growth 2011 9 246 249 10.1016/j.jcrysgro.2010.07.031

[B9] Oh MS Kwon MK Park IK Baek SH Park SJ Lee SH Jung JJ Improvement of green LED by growing p-GaN on In_0.25_GaN/GaN MQWs at low temperature J Cryst Growth 2006 9 107 112 10.1016/j.jcrysgro.2005.10.129

[B10] Ee YK Li XH Biser JE Cao W Chan HM Vinci RP Tansu N Abbreviated MOVPE nucleation of III-nitride light-emitting diodes on nano-patterned sapphire J Cryst Growth 2010 9 1311 1315 10.1016/j.jcrysgro.2009.10.029

[B11] Huang HH Wua YR Light emission polarization properties of semipolar InGaN/GaN quantum well J Appl Phys 2010 9 053112 10.1063/1.3327794

[B12] Cho HK Lee JY Kim CS Yang GM Structural and optical investigation of InGaN/GaN multiple quantum well structures with various indium compositions J Electron Mater 2001 9 1348 1352 10.1007/s11664-001-0123-y

[B13] Moon YT Kim DJ Song KM Kim DW Yi MS Noh DY Park SJ Effect of growth interruption and the introduction of H_2_ on the growth of InGaN/GaN multiple quantum wells J Vac Sci Technol B 2000 9 2631 2634 10.1116/1.1327298

[B14] Choi SB Shim JP Kim DM Jeong HI Jho YD Song YH Lee DS Effect of indium composition on carrier escape in InGaN/GaN multiple quantum well solar cells Appl Phys Lett 2013 9 033901 10.1063/1.4813623

[B15] Jung SH Song KM Choi YS Park HH Shin HB Kang HK Lee J Light output enhancement of InGaN/GaN light-emitting diodes with contrasting indium tin-oxide nanopatterned structures J Nano Mat 2013 9 http://dx.doi.org/10.1155/2013/832170

[B16] Sun G Xu G Ding YJ Zhao HP Liu GY Zhang J Tansu N Investigation of fast and slow decays in InGaN/GaN quantum wells Appl Phys Lett 2011 9 081104 10.1063/1.3627166

[B17] Holec D Costa PMFJ Kappers MJ Humphreys CJ Critical thickness calculations for InGaN/GaN J Cryst Growth 2007 9 314 317 10.1016/j.jcrysgro.2006.12.054

[B18] Horng RH Lin ST Tsai YL Chu MT Liao WY Wu MH Lin RM Lu YC Improved conversion efficiency of GaN/InGaN thin-film solar cells IEEE Electron Device Letters 2009 9 724 726

[B19] Fischer AM Wei YO Ponce FA Moseley M Gunning B Doolittle WA Highly luminescent, high-indium-content InGaN film with uniform composition and full misfit-strain relaxation Appl Phys Lett 2013 9 131101 10.1063/1.4822122

[B20] Yamaguchi T Uematsu N Araki T Honda T Yoon E Nanishi Y Growth of thick InGaN films with entire alloy composition using droplet elimination by radical-beam irradiation J Cryst Growth 2013 9 123 126

[B21] Wang HC Lu YC Chen CY Yang CC Ultrafast pump-probe spectroscopy in the UV-blue range with an extremely broad probe spectrum for the carrier relaxation study in an InGaN thin film with indium-rich nano-clusters Opt Exp 2007 9 3417 3425 10.1364/OE.15.003417 19532583

[B22] Wang HC Lu YC Chen CY Yang CC Carrier capture times of the localized states in an InGaN thin film with indium-rich nano-cluster structures Appl Phys Lett 2006 9 011906 10.1063/1.2219131

[B23] Wang HC Lu YC Chen CY Yang CC Non-degenerate fs pump-probe study on clustered InGaN with multi-wavelength second-harmonic generations Opt Exp 2005 9 5245 5252 10.1364/OPEX.13.005245 19498516

[B24] Chen H Feenstra RM Northrup JE Zywietz T Neugebauer J Greve DW Indium incorporation and surface segregation during InGaN growth by molecular beam epitaxy: experiment and theory MRS Internet J Nitride Semicond Res 2001 9 11

[B25] Feng SW Lai CM Chen CH Sun WC Tu LW Theoretical simulations of the effects of the indium content, thickness, and defect density of the i-layer on the performance of p-i-n InGaN single homo-junction solar cells J Appl Phys 2010 9 093118 10.1063/1.3484040

[B26] Hartono H Chen P Chua SJ Fitzgerald EA Growth of InN and its effect on InGaN epilayer by metalorganic chemical vapor deposition Thin Solid Films 2007 9 4408 4411 10.1016/j.tsf.2006.07.112

[B27] Pereira S Correia MR Pereira E O’Donnell KP Alves E Sequeira AD Franco N Watson IM Deatcher CJ Strain and composition distributions in wurtzite InGaN/GaN layers extracted from x-ray reciprocal space mapping Appl Phys Lett 2002 9 3913 3915 10.1063/1.1481786

[B28] Zhao W Wang L Wang JX Hao ZB Luo Y Theoretical study on critical thicknesses of InGaN grown on (0 0 0 1) GaN J Cryst Growth 2011 9 202 204 10.1016/j.jcrysgro.2011.05.002

[B29] Li Q Westlake KR Crawford MH Lee SR Koleske DD Figiel JJ Cross KC Fathololoumi S Mi Z Wang GT Optical performance of top-down fabricated InGaN/GaN nanorod light emitting diode arrays Opt Exp 2011 9 25528 10.1364/OE.19.025528 22273946

[B30] Wu MH Chang SP Chang SJ Horng RH Liao WY Lin RM Characteristics of GaN/InGaN double-heterostructure photovoltaic cells Int J Photoenergy 2012 9 5 Article ID 206174

[B31] Hsu PS Hardy MT Young EC Romanov AE DenBaars SP Nakamura S Speck JS Stress relaxation and critical thickness for misfit dislocation formation in (10–1 0) and (30-3-1) InGaN/GaN heteroepitaxy Appl Phys Lett 2012 9 171917 1~−4 10.1063/1.4707160

[B32] Wang HC Lu YC Teng CC Chen YS Yang CC Ma KJ Pan CC Chyi JI Ultrafast carrier dynamics in an InGaN thin film J Appl Phys 2005 9 033704-1–4

[B33] Reed MJ El-Masry NA Parker CA Roberts JC Bedair SM Critical layer thickness determination of GaN/InGaN/GaN double heterostructures Appl Phys Lett 2000 9 4121 10.1063/1.1334361

[B34] Choi SK Jang JM Jung WG Kim JY Kim SD Structural and optical properties of InGaN/GaN single quantum well grown via MOCVD Electron Mater Lett 2008 9 67 70

[B35] Inahama S Akiyama T Nakamura K Ito T Theoretical investigation of indium surface segregation in InGaN thin films e-J Sur Sci Nanotech 2005 9 503 506

[B36] Subagio A Sutanto H Supriyanto E Budiman M Arifin P Sukirno S Barmawi M Study of In_0.62_Ga_0.38_N and In_0.62_Ga_0.38_N/GaN single-heterostructure films grown on sapphire substrate by plasma assisted MOCVD method Proceedings of Asian Phys Symposium 2005 Bandung, Indonesia, New York: American Institute of Physics 362 365

[B37] Parker CA Roberts JC Bedair SM Reed MJ Liu SX El-Masry NA Determination of the critical layer thickness in the InGaN/GaN heterostructures Appl Phys Lett 1999 9 2776 2778 10.1063/1.125146

[B38] Zhao HP Liu GY Zhang J Poplawsky JD Dierolf V Tansu N Approaches for high internal quantum efficiency green InGaN light-emitting diodes with large overlap quantum wells Opt Exp 2011 9 A991 A1007 10.1364/OE.19.00A991 21747571

[B39] Zhang J Tansu N Optical gain and laser characteristics of InGaN quantum wells on ternary InGaN substrates IEEE Photo J 2013 9 2600111

[B40] Ishikawa H Nakada N Nakaji M Zhao GY Egawa T Jimbo T Umeno M Investigations on strained AlGaN/GaN/sapphire and GaInN multi-quantum-well surface LEDs using AlGaN/GaN Bragg reflectors IEICE Trans Electron 2000 9 591 597

[B41] Krost A Dadgar A GaN-based optoelectronics on silicon substrates Mat Sci Eng B 2002 9 77 84 10.1016/S0921-5107(02)00043-0

[B42] Shchekin OB Epler JE Trottier TA Margalith T Steigerwald DA Holcomb MO Martin PS Krames MR High performance thin-film flip-chip InGaN–GaN light-emitting diodes Appl Phys Lett 2006 9 071109 10.1063/1.2337007

[B43] Young EC Gallinat CS Romanov AE Tyagi A Wu F Speck JS Critical thickness for onset of plastic relaxation in (1122) and (2021) semi-polar AlGaN hetero-structures Appl Phys Exp 2010 9 111002 10.1143/APEX.3.111002

[B44] Graber A Averbeck R Barnhöfer U Riechert H Tews H Optical characterization of InGaN layers and GaN/InGaN/GaN double heterostructures Mat Sci Forum 1998 9 264 268

[B45] Krames M Watanabe S Shen Y Götz W Müller G Gardner N Chen G Blue-emitting InGaN-GaN double-heterostructure light-emitting diodes reaching maximum quantum efficiency above 200 A/cm^2^ Appl Phys Lett 2007 9 243506 243506 10.1063/1.2807272

[B46] Ambacher O Growth and applications of group III-nitrides J Phys D Appl Phys 1998 9 2653 2710 10.1088/0022-3727/31/20/001

[B47] Wadas TJ Wong EH Weisman GR Anderson CJ Coordinating radiometals of copper, gallium, indium, yttrium and zirconium for PET and SPECT imaging of disease Chem Rev 2010 9 2858 2902 10.1021/cr900325h 20415480 PMC2874951

[B48] Meher SR Biju KP Jain MK Growth of indium-rich nano-crystalline indium gallium nitride thin film by modified activated reactive evaporation Int J Nanosci 2011 9 141 10.1142/S0219581X11007612

[B49] Wang H Wang LL Sun X Zhu JH Liu WB Jiang DS Zhu JJ Zhao DG Liu ZS Wang YT Zhang SM Yang H Suppression of indium droplet formation by adding CCl4 during metalorganic chemical vapor deposition growth of InN films Semicond Sci Tech 2009 9 075004 10.1088/0268-1242/24/7/075004

[B50] Guo Y Liu XL Song HP Yang AL Xu XQ Zheng GL Wei HY Yang SY Zhu QS Wang ZG A study of indium incorporation in In-rich InGaN grown by MOVPE Appl Surf Sci 2010 9 3352 3356 10.1016/j.apsusc.2009.11.081

[B51] Nedeljković JM Mićić OI Ahrenkiel SP Miedaner A Nozik AJ Growth of InP nanostructures via reaction of indium droplets with phosphide ions: synthesis of InP quantum rods and InP-TiO_2_ composites J Am Chem Soc 2004 9 2632 2639 10.1021/ja039311a 14982473

[B52] Lu HQ Thothathiri M Wu Z Bhat I Study of indium droplets formation on the In_x_Ga_1 − x_N films by single crystal x-ray diffraction J Electron Mater 1997 9 281 284 10.1007/s11664-997-0164-y

[B53] Chen YS Liao CH Cheng YC Kuo CT Wang HC Nanostructure study of the coalescence growth of GaN columns with molecular beam epitaxy Opt Mat Exp 2013 9 1450 1458 10.1364/OME.3.001450

[B54] Wu F Tyagi A Young EC Romanov AE Fujito K DenBaars SP Nakamura S Speck JS Misfit dislocation formation at heterointerfaces in (Al, In)GaN heteroepitaxial layers grown on semipolar free-standing GaN substrates J Appl Phys 2011 9 033505 10.1063/1.3531577

[B55] Sánchez AM Gass M Papworth AJ Goodhew PJ Singh P Ruterana P Cho HK Choi RJ Lee HJ V-defects and dislocations in InGaN/GaN heterostructures Thin Sol Film 2005 9 316 320 10.1016/j.tsf.2004.11.207

[B56] Chen YS Liao CH Chueh YL Kuo CT Wang HC Plan-view transmission electron microscopy study on coalescence overgrowth of GaN nano-columns by MOCVD Opt Mat Exp 2013 9 1459 1467 10.1364/OME.3.001459

[B57] Yoon DH Lee KS Yoo JB Seong TY Reduction of threading dislocations in InGaN/GaN double heterostructure through the introduction of low-temperature GaN intermediate layer Jpn J Appl Phys 2002 9 1253 1258 10.1143/JJAP.41.1253

[B58] Koslow IL Hardy MT Hsu PS Wu F Romanov AE Young EC Nakamura S DenBaars SP Speck JS Onset of plastic relaxation in semipolar (11–22) In_x_Ga_1 − x_N/GaN heterostructures J Cryst Growth 2014 9 48

[B59] Ploch S Wernicke T Frentrup M Pristovsek M Weyers M Kneissl M Indium incorporation efficiency and critical layer thickness of (2021) InGaN layers on GaN Appl Phys Lett 2012 9 202102 10.1063/1.4767336

[B60] Kangawa Y Ito T Kumagai Y Koukitu A Kawaguchi N Influence of lattice constraint from InN and GaN substrate on relationship between solid composition of In_x_Ga_1-x_N film and input mole ratio during molecular beam epitaxy Jpn J Appl Phys 2003 9 42

[B61] Singh R Doppalapudi D Moustakas TD Romano LT Phase separation in InGaN thick films and formation of InGaN/GaN double heterostructures in the entire alloy composition Appl Phys Lett 1997 9 1089 1091 10.1063/1.118493

[B62] Gmili YE Orsal G Pantzas K Ahaitouf A Moudakir T Gautier S Patriarche G Troadec D Salvestrini JP Ougazzaden A Characteristics of the surface microstructures in thick InGaN layers on GaN Opt Mat Exp 2013 9 1111 1118 10.1364/OME.3.001111

[B63] Hu FR Kanamori Y Ochi K Zhao Y Wakui M Hane K A 100 nm thick InGaN/GaN multiple quantum-well column-crystallized thin film deposited on Si(111) substrate and its micromachining Nanotechnology 2008 9 035305 10.1088/0957-4484/19/03/035305 21817568

[B64] Tourbot G Bougerol C Glas F Zagonel LF Mahfoud Z Meuret S Gilet P Kociak M Gayral B Daudin B Growth mechanism and properties of InGaN insertions in GaN nanowires Nanotechnology 2012 9 135703 10.1088/0957-4484/23/13/135703 22418250

[B65] Amari H Ross IM Wang T Walther T Characterization of InGaN/GaN epitaxial layers by aberration corrected TEM/STEM Phys Status Solidi C 2012 9 546 549 10.1002/pssc.201100500

